# Caudwell Xtreme Everest: A prospective study of the effects of environmental hypoxia on cognitive functioning

**DOI:** 10.1371/journal.pone.0174277

**Published:** 2017-03-27

**Authors:** Konstadina Griva, Jan Stygall, Mark H. Wilson, Daniel Martin, Denny Levett, Kay Mitchell, Monty Mythen, Hugh E. Montgomery, Mike P. Grocott, Golnar Aref-Adib, Mark Edsell, Tracie Plant, Chris Imray, Debbie Cooke, Jane Harrington, Maryam Khosravi, Stanton P. Newman, for the Caudwell Xtreme Everest Research Group

**Affiliations:** 1 Health Services Research Group, City University London, College Building Room A224, London, United Kingdom; 2 Department of Psychology, National University of Singapore, 9 Arts Link AS4-02-28, Singapore, Singapore; 3 The Traumatic Brain Injury Centre, Imperial College, London and London’s Air Ambulance (Queen Mary University), London, United Kingdom; 4 Institute for Human Health and Performance, University College London, London, United Kingdom; 5 Anaesthesia and Critical Care Research Unit, University Hospital Southampton, Southampton, United Kingdom; 6 Integrative Physiology and Critical Illness Group, Division of Clinical and Experimental Science, Faculty of Medicine, University of Southampton, Southampton, United Kingdom; 7 Southampton NIHR Respiratory Biomedical Research Unit, Southampton, Southampton, United Kingdom; 8 Division of Psychiatry, University College London, London, United Kingdom; 9 St George's Hospital, London, United Kingdom; 10 MRC Centre for Inflammation Research, The Queen's Medical Research Institute, The University of Edinburgh, Edinburgh, United Kingdom; 11 Division of Translational and Systems Medicine, Warwick Medical School, Warwick, United Kingdom; 12 School of Health Sciences, Faculty of Health & Medical Sciences, University of Surrey, Surrey, United Kingdom; Institute of Health Science, CHINA

## Abstract

**Background:**

The neuropsychological consequences of exposure to environmental hypobaric hypoxia (EHH) remain unclear. We thus investigated them in a large group of healthy volunteers who trekked to Mount Everest base camp (5,300 m).

**Methods:**

A neuropsychological (NP) test battery assessing memory, language, attention, and executive function was administered to 198 participants (age 44.5±13.7 years; 60% male). These were studied at baseline (sea level), 3,500 m (Namche Bazaar), 5,300 m (Everest Base Camp) and on return to 1,300 m (Kathmandu) (attrition rate 23.7%). A comparable control group (n = 25; age 44.5±14.1 years; 60% male) for comparison with trekkers was tested at/or near sea level over an equivalent timeframe so as to account for learning effects associated with repeat testing. The Reliable Change Index (RCI) was used to calculate changes in cognition and neuropsychological function during and after exposure to EHH relative to controls.

**Results:**

Overall, attention, verbal ability and executive function declined in those exposed to EHH when the performance of the control group was taken into account (RCI .05 to -.95) with decline persisting at descent. Memory and psychomotor function showed decline at highest ascent only (RCI -.08 to -.56). However, there was inter-individual variability in response: whilst NP performance declined in most, this improved in some trekkers. Cognitive decline was greater amongst older people (r = .42; p < .0001), but was otherwise not consistently associated with socio-demographic, mood, or physiological variables.

**Conclusions:**

After correcting for learning effects, attention, verbal abilities and executive functioning declined with exposure to EHH. There was considerable individual variability in the response of brain function to sustained hypoxia with some participants not showing any effects of hypoxia. This might have implications for those facing sustained hypoxia as a result of any disease.

## Introduction

Tissue hypoxia occurs in response to a diverse range of acute and chronic disease conditions [1] and may impact on neurocognitive outcome [2]. Mild to moderate perioperative hypoxaemia is also implicated in the pathogenesis of postoperative cognitive impairment [3], and cognitive impairment is recognised in those suffering conditions associated with chronic hypoxaemia, such as chronic obstructive pulmonary disease and obstructive sleep apnea [4]. However, apparently normal cognition (as evidenced by effective completion of tasks) is possible in the face of profound hypoxaemia [5] while the role of confounding factors and preexisting vulnerabilities (such as older age, comorbidity, and/or frailty) in the pathogenesis of cognitive decline cannot be completely elucidated in studies involving patient populations.

Research on healthy volunteers offers the opportunity to explore the direct effects of hypoxia on cognition and to identify pathways and avenues for neuroprotection [6,7,8]. Prior work has been instrumental in delineating the cognitive domains most affected [9] yet limited by small sample sizes, restricted ranges of neuropsychological tests, and lack of control groups and/or of serial assessments [8, 10, 11, 12, 13, 14, 15]. Importantly, improvements in neuropsychological test performance, which occur with repeat testing (“practice effects”) can mask declines in cognitive functioning [16, 17, 18] and such effects are rarely adjusted for.

As part of the Caudwell Extreme Everest Medical Research Expedition (February 2007 –June 2007), we compared cognitive function in a large sample of healthy participants exposed to environmental hypobaric hypoxia (EHH) during a trek to the base camp of Mount Everest with a comparator group assessed at the same time points at or near sea level [19, 20]. We also sought to identify trajectories of cognitive function across ascent and examined the associations between cognitive performance and concurrently assessed physiological parameters.

## Methods

### Participants

Participants were recruited from the general public. Inclusion criteria were: age over 18; fluent English speaker; lowland resident; non-professional trekker status; availability for baseline testing; and good physical health (determined by two health-screening tests). Excluded were those with recent (i.e., 3 months pior to departure) altitude exposure (e.g. trekking, skiing) [20]. To estimate the magnitude of practice effects, we used the same inclusion criteria to enroll a group of control participants who did not ascend to altitude. Control participants were recruited opportunistically (snowball sampling [21]) through network of acquaintances, co-workers of trekkers and researchers.

Written informed consent was obtained from all participants. The University College London Research Ethics Committee approved all study procedures (in accordance with the Declaration of Helsinki).

### Experimental protocol/course of expedition

A detailed ascent profile is provided in “Design and Conduct of Caudwell Xtreme Everest: An Observational Cohort Study of Variation in Human Adaptation to Progressive Environmental Hypoxia” [18]. In brief, all participants were initially studied at sea level in London, UK (75 m), and then followed an identical 11-day ascent route to Everest Base Camp (EBC). Subjects were assessed at 3500 m (Namche), 5300 m (EBC) and at 1300 m (upon return to Kathmandu). Laboratory altitudes, barometric pressures and inspired partial pressures of oxygen are summarised in Table 1. The ascent rate was chosen to minimise the incidence of high altitude illness and therefore maximise the number of participants able to contribute data.

**Table 1:** Laboratory altitude, mean barometric pressure, mean laboratory temperature & inspired partial pressure of oxygen.

	*Appx Altitude metres*	*Ambient Temperature ˚C*	*Barometric Pressure mmHg*	*PiO*_*2*_ *mmHg*
**LONDON**	75	24.1 (1)	754 (10)	148.0
**NAMCHE**	3500	19.6 (2.6)	505(3)	95.4
**EBC**	5300	21.5 (5.6)	404 (3)	74.7
**KATHMANDU**	1300	26.1 (1.5)	650 (3)	126.2

Each subject underwent neurocognitive assessment consistently on either day 1 or day 2 after arrival at each point. The control group underwent neuropsychological testing at sea level at the same time intervals as the trekkers.

### Measures

Self-reported demographical data (age, gender, education) were recorded. Resting diastolic and systolic blood pressure (DBP/SBP [mmHg]; Omron M7, Bannockburn, IL, USA), heart rate [HR, bpm], Near Infrared Spectroscopy of cerebral frontal lobes bilaterally (NIRS–rSO_2_/% Invos Cerebral Oximeter 5100C, Somanetics, Troy, MI, USA), haemoglobin concentration in venous blood (g/l) (Hemocue AB, Hemocue, Sweden), percentage saturation of arterial haemoglobin with oxygen (SaO_2_ (%), Nonin Onyx 9500, Nonin Medical Inc, MN, USA), maximal exertional oxygen consumption (ml/kg/min via cardio-pulmonary exercise testing; Metamax 3b, Cortex, Leipzeig, Germany) and anthropometric indices (weight (kg); height (cm)) were recorded at all assessments except for last assessment at Kathmandu. See [19] for details.

#### Cognitive/neuropsychological tests and mood assessments

The neuropsychological assessment test battery was assembled to include all major cognitive domains–attention, memory, executive function, and language–using standardised and commonly used tests in clinical and research settings [22]: Trail Making Test parts A and B [23], Controlled Oral Word Association Test [24], Letter Cancellation Test [22, 25], Stroop Test [26], Grooved Pegboard [27], Rey Auditory Verbal Learning Test [28], Symbol Digit Modalities Test [29] and Block Design Test [30] (see Table 2 for description). Alternate forms of the tests were used when available [22]. Tests were administered in a fixed order by trained research personnel. Intelligence (IQ) testing (using the Wechsler Intelligence Test [30]) was only performed at baseline.

**Table 2:** Description of Neuropsychological Tests.

NP Tests	Cognitive domains	Test description	Performance measures
Wechsler Intelligence Test	General intelligence	Battery of verbal and performance based tasks to provide IQ scores	Full IQ score; Verbal IQ score; Performance IQ score
Trail Making Test Part A (TMT-A)^1^	Attention / concentration	Connect 25 consecutive numbers in correct order as fast as possible	Time to completion (s)
Trail Making Test Part B (TMT-B)^1^	Attention and executive function	Efficiently alternate between sequential list of numbers & letters	Time to completion (s)
Symbol Digit Modality Test^1^ (SDMT)	Attention / concentration	Visual scanning & rapid written response in substituting symbols for numbers according to learned code	No correct substitutions within 90 seconds
Letter Cancellation Test (LCT)	Attention / visual scanning and mental speed	Ability to visual scan & cancel designated letters in working sheets of randomly allocated letters as fast as possible	Time to completion
Block Design (BD)	Visuo-spatial and motor skills; non-verbal problem solving	Ability to reproduce designs of increasing difficulty using blocks	Time to completion
Rey Auditory Verbal Learning Test^1^	Learning and verbal memory	Ability to retain and recall words	Total of correct recalls across 5 trials (verbal learning score) (RAVLT-L). Total words recalled after distraction (delayed recall score) (RAVLT-D)
Stroop Color Word Test	Attention and executive function	Part 1: To read color words and disregard color ink. Part 2. To name the color in which the color names are printed and disregard their verbal content	Time to completion (Part 2; color word task)
Controlled Oral Word Association^1^ (COWA)	Verbal ability / verbal fluency	Ability to make verbal associations to specified letters	Total of correct words beginning with the designated letters over 1 minute trials
Grooved Pegboard	Psychomotor speed and manual dexterity	Ability to perform the task of picking and placing pegs onto board varying in orientation using 1 hand	Time to completion in seconds (dominant (GP-D) and non-dominant (GP-ND) hands measured separately)

Note

^1^ Alternate forms used

The Brief Center for Epidemiologic Studies Depression Scale [31] and State Trait Anxiety Inventory [32] measured depression and anxiety symptoms respectively. Symptoms of Acute Mountain Sickness (AMS) were evaluated using the Lake Louise Symptoms Score [33].

### Statistical analysis

In order to assess change at a group level, cognitive outcomes were compared between baseline and follow-up assessments for the trekker and control groups using a series of mixed factor analyses of variance. Three analyses were performed. The first compared group differences in changes of performance of each of the neuropsychological tests. The second and most critical analyses utilized the scores of the control group in constructing a practice adjusted Reliable Change Index (RCI) method [34, 18] to adjust individual differences in test scores for measurement error and practice effects. RCIs were determined by subtracting the baseline score (X_1_) from the follow up scores (X_2_), giving (DELTA)_X_ for each individual participant for a given task. The mean measured change for the controls, (DELTA)_Xc_, calculated in the same way, was then subtracted from this, removing any practice effect. This score was then divided by the within-subject standard deviation for control group, controlling for expected variability. This formula gives a precise estimate of relative change controlling for variability and practice–it is comparable with various regression based estimates and clearly superior to simple change scores in ability reliable and clinically meaningful change [18]

These RCI scores were then used to create individual and combined test scores (Z_combined_) using the sum of standardized RCI scores (z RCI) for each test divided by the standard deviation of this summation in the control group. This technique identifies cognitive change by comparing the changes in test scores of an individual trekker with changes in the test scores of the control group over the same interval. The sign was adjusted so that negative z scores indicated deterioration from the baseline test. In the third analysis, the RCI scores were examined for each individual to determine the percentage of individuals who demonstrated a decline in performance for each test (i.e., negative RCI).

To further examine patterns of cognitive performance, Latent Profile Analysis (LPA) was conducted on total/combined RCI scores in Mplus Version 6.12 [34]. An increasing number of classes were fit in a series of iterative steps until the resultant model was not well identified [35]. The optimal number of classes was determined using the Akaike Information Criterion (AIC) [36], Bayesian Information Criterion (BIC) [37], and the Bootstrapped Likelihood Ratio Test (BLRT) [38, 39]. We sought a model with lower values for the information criteria, and *p* values ≤ .05 for the likelihood ratio test. In addition, classification quality was indicated by higher relative entropy (≥ .80), average posterior class probability (≥.70), and odds of correct classification (≥ 5) [40, 41,42]. The associations between cognitive change indices (individual RCI; and trajectories of total RCI scores) to demographic and clinical parameters were explored using correlations or ANOVA (as appropriate). For physiological parameters, both absolute levels as well the percentile change at each ascent point relative to baseline were employed.

To control for missing data, we repeated all analyses using all cases enrolled at baseline after missing data imputation (last value carried forward approach) as a sensitivity analysis. Significance levels were set at p < .05. All values reported are mean ± SD unless stated otherwise.

## Results

One hundred and ninety-eight trekker participants (44.7±13.7 years; 63% male; years of full time education = 17.1±2.3; Weight (kg) = 79.99±13.52; Height (cm) = 172.68±9.18) and 25 control participants (44.5±14.1 years; 60% male; years of full time education = 16.8±2.9) were studied. Trekker and control groups were comparable in their demographic characteristics (p > .05 in all cases). Complete cognitive data across all four assessments were obtained in N = 153 trekkers (attrition rate = 23.7%) with data missing from N = 12 at Namche, N = 23 at EBC, and N = 48 on return to Kathmandu. Reasons for missing data included poor health/injury or tiredness (N = 33), logistic difficulties such as weather conditions or delayed flights (N = 42), withdrawal (N = 1) and inability to complete for other reasons such as preoccupation with other concerns (N = 7).

The characteristics and baseline neuropsychological performance of the trekkers who provided data for analysis of cognitive outcomes across all four assessments were comparable to those of the total sample and the non-completers (p > .05 in all cases), indicating that the missing data did not reflect a selective loss to follow up. Details of physiological parameters and symptoms across ascent are depicted in Table 3.

**Table 3:** Symptoms and physiological measurements across assessments.

	London (sea level)	Namche	EBC	Kathmandu
	M (SD)	M (SD)	M (SD)	M (SD)
**Resting SPB_+_**	1.02 (20.33)	136.63 (17.41)	147.08 (20.14)	
**Resting DSB_+_**	78.90 (10.45)	85.04 (10.06)	89.52 (10.60)	
**Heart Rate (HR, bpm) _1_**	71.48 (13.58)	74.85 (12.86)	76.09 (13.85)	
**Hemoglobin (Hb, g/l) _1_**	144.78 (12.46)	147.23 (13.20)	157.67 (13.58)	
**SaO_2_ (%)_1_**	97.60 (1.96)	88.61 (3.72)	77.70 (5.26)	
**NIRS–rSO_2_/%_1_**	68.57 (8.35)	58.80 (7.44)	53.68 (7.32)	
**Maximal exertional oxygen consumption (ml/kg/min)**	.375 (.077)	.394 (.079)	.403 (.082)	
**LLS_2_**		2.31 (2.26)	2.93 (2.40)	
**Anxiety**	30.84 (8.06)	32.25 (8.53)	34.05 (10.87)	25.64 (6.78)
**Depression**	7.12 (6.48)	7.77 (5.68)	11.32 (7.72)	9.19 (7.19)

Note: SBP, systolic blood pressure; DPB, diastolic blood pressure; HR, heart rate; NIRS, Near Infrared Spectroscopy; SaO_**2,**_ percentage saturation of arterial haemoglobin with oxygen; Hb, Hemoglobin; LLS, Lake Louise Symptoms Score.

_1_ Physiological measurements were not taken at descent to Kathmandu.

_2_ LLS was not taken at baseline and descent to Kathmandu.

### Group comparisons on cognition

Mean neuropsychological scores for both trekkers and control groups were within the reference range for all NP tests (within one standard deviation (SD) of general population test norms) [43] (see Table 4 for mean scores).

**Table 4:** Absolute NP scores (Mean and Sd) across ascent & descent points for trekkers and controls.

	BASELINE		NAMCHE		EVEREST BASE CAMP		KATHMANDU	
	Trekkers	Controls	Trekkers	Controls	Trekkers	Controls	Trekkers	Controls
TMT A^1^	24.68 (7.72)	29.44 (7.20)	27.28 (7.78)	28.10 (7.95)	21.98 (6.75)	25.40 (8.17)	20.34 (6.29)	21.95 (5.12)
TMT B^1^	50.17 (17.43)	57.80 (19.63)	50.85 (14.37)	49.10 (12.25)	48.66 (16.99)	49.05 (16.63)	41.43 (18.42)	46.05 (16.57)
COWA^2^	45.56 (12.28)	44.64 (9.05)	42.95 (12.14)	44.95 (11.16)	47.61 (13.89)	50.60 (11.39)	48.06 (12.13)	52.25 (12.38)
LCT^1^	72.12 (17.73)	80.32 (16.96)	86.92 (18.40)	86.86 (17.34)	76.66 (18.06)	79.00 (15.49)	76.94 (21.63)	74.65 (14.79)
Stroop CW^1^	114.88 (25.89)	116.36 (29.94)	108.37 (22.25)	102.52 (21.73)	104.08 (22.86)	95.95 (21.59)	102.06 (22.05)	95.15 (16.68)
GP D^1^	65.10 (12.55)	62.84 (10.19)	61.32 (10.45)	61.90 (10.12)	62.51 (10.93)	59.75 (11.22)	59.43 (10.34)	57.55 (8.71)
GP ND^1^	70.81 (12.55)	70.08 (12.17)	65.30 (10.60)	66.15 (11.01)	69.11 (12.06)	65.32 (12.46)	65.69 (12.55)	64.05 (10.07)
RAVLT- T^2^	54.05 (8.22)	59.72 (9.48)	59.58 (8.97)	64.95 (9.18)	57.24 (9.32)	63.70 (10.34)	57.87 (10.08)	63.40 (12.71)
RAVLT- D^a^	-1.45 (1.86)	-2.16 (2.23)	-.95 (1.69)	-.43 (.75)	-1.67 (1.96)	-1.40 (1.98)	-1.69 (2.09)	-2.15 (1.87)
Symbol Digit^2^	58.79 (10.06)	56.64 (10.07)	59.57 (11.39)	61.38 (8.95)	62.09 (13.15)	62.95 (10.59)	64.10 (14.27)	66.25 (11.85)
Block Design^2^ ^3^	39.86 (8.37)	34.80 (10.65)	-	-	40.99 (8.54)	39.70 (8.11)	42.39 (10.08)	41.20 (8.82)

Note: TMT A, Trail Making Test, Form A; TMT B, Trail Making Test, Form B; LCT, letter cancellation test; SDTM–W, Symbol Digit Modality Test written administration; RAVLT–T, Rey Auditory Verbal Learning Test total word recall after Trials 1–5; RAVLT–D, Rey Auditory Verbal Learning Test drop in retention from Trials 5 to 7; GP–DOM, Grooved Pegboard dominant hand; GP–NDOM, Grooved Pegboard non dominant hand.

^1^ Time to completion in seconds (i.e. higher scores worse NP performance)

^2^ Number correct (i.e. higher scores better NP performance)

^3^ Drop in number of words recalled (i.e. higher scores worse NP performance).

^a^ The Block Design was not performed in Namche.

Trekkers and controls had equivalent NP scores with the exception of RAVLT-Total, where control subjects outperformed trekkers on all assessments including baseline. Generally, mean cognitive performance across NP tests was significantly better from baseline to follow-up assessments in both trekkers and controls, albeit not uniformly for all tests, providing evidence of the expected practice effects with repeated NP administrations over short time intervals (*p* values < .05). Post-hoc tests showed that performance improvements over repeat assessments were greater in the control group relative to trekkers where slopes were less steep but still significant. As expected, evidence of a significant decline in raw NP scores at the group level was not revealed on any of the neuropsychological tests for either group.

### Course of cognition using reliable cognitive change index (RCI)

Data are summarized in Table 5. Controlling for practice effects using RCI methodology demonstrated an altitude-related decline in cognitive performance for the trekkers. Mean RCI scores revealed cognitive decline with ascent for all tasks except for the GP-D, GP-ND and RAVLT-L, which only deteriorated at the highest altitude (i.e., Everest Base Camp) (Table 4). Inspection of RCI values indicated that there was a greater decline for most of tests at the first ascent point (Namche). Decline was noted in all tests at EBC and persisted on return to Kathmandu for all domains but for GP-D, GP-ND and RAVLT-L. Decline was more pronounced in tests of verbal ability/language (e.g. COWA; LCT) and executive function (e.g. BD; TMT-B) whereas test of psychomotor function and verbal memory were only affected at highest ascent. Sensitivity analyses using missing data imputation indicated a similar pattern of decline as that identified in the primary analysis above.

**Table 5:** Group mean (SD) and % of trekkers showing cognitive decline on the RCI individual and summary neuropsychological scores.

	Namche		EBC		Kathmandu	
	M (SD)	% Trekkers decline	M (SD)	% Trekkers decline	M (SD)	% Trekkers decline
**TMT A^1^**	-.69 (1.38)	72.3%	-.16 (1.30)	64.8%	-.41 (.82)	74.4%
**TMT B^1^**	-.80 (1.42)	79.8%	-.63 (.155)	74.2%	-.05 (.79)	58.2%
**COWA^2^**	-.72 (1.76)	69.5%	-.72 (1.45)	74.6%	-.83 (1.17)	76.9%
**LCT^1^**	-.75 (1.66)	72.8%	-.54 (1.59)	67.1%	-.95 (1.48)	76.1%
**Stroop^1^**	-.17 (1.15)	62.4%	-.24 (.99)	66.2%	-.30 (.90)	67.3%
**GP D^1^**	1.13 (2.61)	38%	-.15 (2.29)	56.8%	.12 (1.74)	49.1%
**GP ND^1^**	.56 (1.36)	38%	-.32 (2.44)	64.8%	.24 (1.71)	37.3%
**RAVLT-L^2^**	.06 (1.64)	46.5%	-.08 (1.79)	60.6%	.14 (1.46)	44.7%
**RAVLT-D^3^**	-.48 (1.42)	73.2%	-.56 (2.39)	73.2%	-.47 (1.91)	61.6%
**Symbol Digit^2^**	-.60 (1.32)	80.8%	-.14 (1.29)	63.4%	-.43 (1.03)	70%
**Block Design^2^^a^**	-	-	-.87 (1.06)	84.5%	-.74 (.95)	83.7%
**Total RCI ^a^**	-.25 (.62)	76.1%	-.36 (.71)	77%	-.27 (.49)	69.3%

Note: TMT A, Trail Making Test, Form A; TMT B, Trail Making Test, Form B; LCT, letter cancellation test; SDTM–W, Symbol Digit Modality Test written administration; RAVLT–T, Rey Auditory Verbal Learning Test total word recall after Trials 1–5; RAVLT–D, Rey Auditory Verbal Learning Test drop in retention from Trials 5 to 7; GP–DOM, Grooved Pegboard dominant hand; GP–NDOM, Grooved Pegboard non dominant hand.

^1^ Time to completion in seconds

^2^ Number correct

^3^ Drop in number of words recalled.

^a^ The block design was not performed in Namche so the total RCI scores were calculated without the block design at all assessment points.

### Incidence of cognitive decline

Individual variability in performance was assessed by classifying individual participant RCIs as either declined or not declined on each of the individual NP tests. With practice effects accounted for, between two thirds and three quarters of trekkers demonstrated decline in individual NP tests (see Table 4 above; see S1 Fig and S2 Fig). The mean number of test scores showing decline was comparable across ascent points but varied across trekkers. The mean number of test scores showing a decline was 5.38±2.28 (range = 10) in Namche; at EBC the mean was 6.23± 2.49 test scores (range = 9) whereas in Kathmandu, decline was noted in 5.15±3.42 (range = 11) NP test scores.

**Fig 1:**
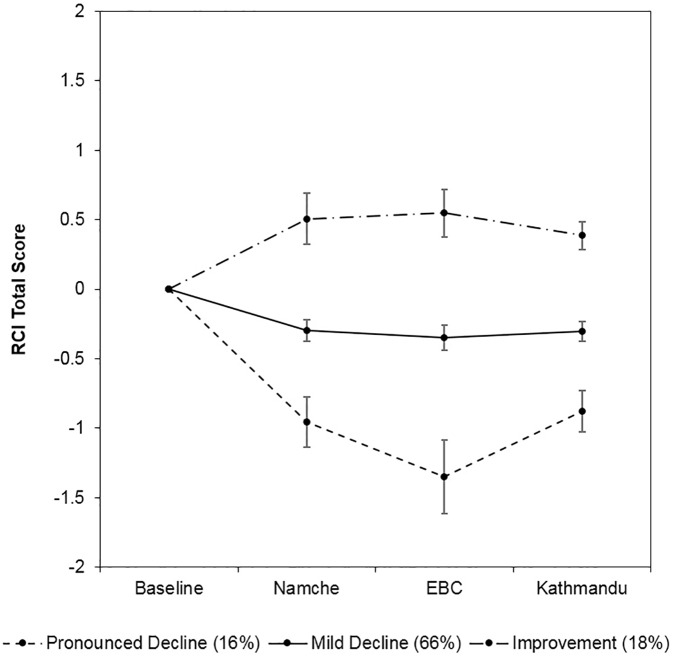
Latent profiles of RCI total scores among trekkers. Error bars represent 95% confidence intervals.

The latent profile analysis of summary NP scores indicated similar decline rates. In this analysis, one- to three-class solutions were fit in a series of iterative steps to determine the optimal number of profiles (the four-class solution was not well-identified). Model fit improved as the number of classes increased (Table 6). As a result, the three-class solution was selected. This solution demonstrated good relative entropy (.82), average posterior class probabilities (.91–.93) and odds of correct classification (7.23–48.49). The three distinct profiles of cognitive function among trekkers are shown in Fig 1: pronounced decline (16%; N = 29), mild decline (66%; N = 118) and improvement (18%; N = 33).

**Table 6:** Fit indices for latent profile analysis on RCI total scores.

	LL ^a^	AIC ^b^	BIC ^c^	Entropy	BLRT ^d^ (*p* value)
1 Class	-449.00	909.98	929.14	–	–
2 Class	-402.49	824.98	856.91	.65	93.00 (< .001)
3 Class	-367.62	763.24	807.94	.82	69.74 (< .001)

Note

^a^ Log-likelihood

^b^ Akaike Information Criterion

^c^ Bayesian Information Criterion

^d^ Bootstrapped Likelihood Ratio Test.

### Factors related to cognitive decline

Associations with demographic and clinical parameters were examined separately at each assessment point (for individual and summary RCI scores) and in relation to overall cognitive trajectories across expedition (mild decline; pronounced decline; improvement).

The gender, education, anthropometric indices (height; weight) and mood (anxiety and depression symptoms) of the group of participants who exhibited cognitive decline did not differ from those who exhibited no such decline. There were a few significant associations between RCI scores and age, education and mood albeit not consistently for all NP tests and across all ascent points. Any significant associations of mood and education with cognitive decline were mainly found at the highest point of ascent (EBC) but not for all cognitive tests. Anxiety and depression were in the main unrelated to cognitive decline with only a handful of significant (small-sized) associations between symptoms of anxiety with decline in memory (r = -.16; p = .035 for RAVLT-T and r = -.15; p = .043 for RAVLT-D) and overall total RCI summary scores at EBC (r = -.17 p = .032). Symptoms of depression correlated only with decline in one NP test, namely SD at EBC (r = -.19; p = .011).

The number of years of full time education was found to be inversely associated with decline in two of the 11 cognitive test scores, i.e. TMT-A (r = -.20; p = .012), STROOP (r = -.28; p = .001), at EBC. Age, on the other hand, was significantly associated with decline in TMT-A (r = -.19; p = .015),; TMT-B (r = -.19; p = .014); LCT (r = -.46; p < .0001); SDMT (r = -.29; p < .0001); BD (r = -.24; p = .003); and total RCI scores (r = -.27; p = .005) and count of tests showing decline (r = .42; p < .0001) but only at the last assessment point, i.e., return back to Kathmandu. The greater decline with age on descent to Kathmandu suggests that cognitive recovery may vary as a function of age.

The symptoms of AMS were associated with decline in some tests, but only at EBC. These tests were: RAVLT-D (r = -.19; p = .011); COWA (r = -.20; p = .008); STROOP (r = -.15; p = .045) and Total RCI Summary Scores (r = -.25; p = .001). IQ scores and physiological parameters, i.e. SPB, NIRS, SaO_2_ (absolute or percentile change relative to baseline) were unrelated to cognitive decline (p>.05 in all cases). The only physiological variable with significant (albeit inconsistent) associations with cognitive assessments was DPB. Increased DBP (percentile change relative to baseline) was significantly associated with decline in COWA (r = -.16; p = .032) at Namche and decline in SD (r = -.15; p = .038) and BD (r = -.15; p = .039) at EBC.

Analyses on cognitive profiles indicated no significant differences between the three trajectories (mild decline; pronounced decline; improvement) in any of the socio-demographic, physiological or psychological variables examined.

## Discussion

This is the largest prospective study to investigate the impacts of environmental hypoxia on cognitive ability. As expected, absolute cognitive performance improved with experience of repeated testing in both trekkers and controls. Adjustment for such ‘practice effects’, using RCI methodology and profiling of cognitive trajectories across the expedition, provided clear evidence of overall cognitive impairment across several domains in the group of trekkers exposed to altitude and environmental hypoxia. Cognitive declines were evident in the group on all NP tasks at the highest point of ascent, i.e. Everest Base Camp (5,300 m). Of note, whilst cognitive performance improved on descent from Everest Base Camp to Kathmandu (1,300m), it remained impaired when compared to pre-trek levels and even to those recorded at greater altitude (Namche: 3,500m) on ascent. Thus, return to lower altitude does not immediately restore the cognitive effects of exposure to hypoxia. Age was unrelated to RCI-based NP tests or summary scores at high altitudes (both Namche and Everest Base Camp) but was significant on descent to Kathmandu suggesting poorer cognitive recovery in older participants—although whether this persists is not known. This finding is in keeping with other studies where the vulnerability of the cognitive functioning of older people has been found to be associated with poorer recovery in a range of conditions including traumatic brain injury and surgery [44, 45].

Cognitive decline was greatest in executive function, complex attention, and verbal skills [8, 9, 46, 47, 48, 49]. There was a small improvement in psychomotor abilities at 1,300 m (Kathmandu) and 3,500 m (Namche) when compared at baseline, but this declined at the highest altitude (EBC). Verbal memory did not decline at first ascent testing (Namche; 3,500 m) yet declines in memory were found at all subsequent testing points. The notion of a threshold may explain the lack of decline in motor function and memory at altitudes lower than 4,000 m. Motor precision skills reflected in GP are thought to be affected at higher altitudes in comparison to motor speed [12]. It has been suggested that a distinct pattern of neuropsychological change develops above 4,000 m altitude and this is consistent with the cognitive decline in all cognitive domains at the highest point of ascent (Everest Base Camp at 5,300 m). These findings are in line with the proposed transitional zone at 4000 m for loss of autoregulation in cerebral blood flow [50]. Variation in the susceptibility to hypoxia of different brain regions (i.e. hippocampus, basal ganglia, cerebellum and occipital cortex), each associated with particular cognitive functions, may also account for the observed pattern of results [51]. An alternative explanation may relate to differences in test sensitivity and levels of difficulty. Grooved Pegboard, a test of motor precision, may be considered a less complex task relative to the tests of attention and executive function deployed in this study. These more complicated tasks showed deterioration at lower altitudes. The observed declines in cognitive performance at the lower altitudes cannot be attributed to AMS. AMS symptoms were related to decline in verbal abilities and executive function only at highest point of ascent (5,300) but not at lower altitudes. There was also no clear physiological pathway as none of measures taken showed reliable associations across tests or testing to allow some confidence of a plausible physiological mechanism.

While cognitive decline was demonstrated for most trekkers, some were unaffected or even performed better relative to baseline after practice effects were accounted for. However, decline with altitude was the norm for most. This pattern was shown both in analyses of individual tests as well as overall cognitive profiles across ascent. Such variability is consistent with that observed in other physiological responses to high altitude. The reasons for such variation remain unclear. With the exception of differential rates of recovery on descent being related to age, variability in performance was not consistently explained by socio-demographic characteristics (age, education, smoking), cognitive reserve (i.e., IQ), physiological parameters such as oxygen saturation levels or time intervals between assessments. It is of note that NP decline in some tests and on the overall measure of NP was associated with the symptoms of AMS only at the highest level of ascent. It is possible that the threshold for symptoms of AMS at that point is of sufficient severity to have a generalised effect including on cognition.

The heterogeneity in cognitive outcomes in individual performance represents a challenge for prediction in conditions of hypoxia. This is in common with a growing but incomplete understanding of the mechanisms underlying cognitive decline, resilience or recovery. Whilst hypoxia itself appears the most cogent explanation of the overall results, the variability can be attributed to factors such as inflammatory processes, oxidative stress, cortical damage, cumulative effects of repeated ascents, prolonged altitude exposure, immune alterations and fatigue, sleep disturbances, or psychological stressors [52]. All of these possibilities are highly speculative at this point, but generate potential hypotheses for future research on individual differences apparent in these results.

A particular strength of the current study is our sample size (the largest to date for such a study) that allowed the statistical modeling of trajectories of cognitive change; the use of controls matched for age and educational background, so as to control for measurement error and practice effects; and the use of a comprehensive cognitive battery. Most other studies have used small samples (that precluded statistical modeling) and limited neuropsychological tests. The current study adds to the literature in two important ways. First, we have used to model trajectories of cognitive test scores over incremental exposure to hypobaric hypoxia. The results of these analyses reveal that there is heterogeneity in the profiles of change over time in a large sample av. Second, these results also help to tease apart the relative contributions of maturation (i.e., normal aging) and practice effects that can affect follow-up test scores.

Our study does, however, have weaknesses. Limitations in carrying equipment to altitude, and unreliability of power supply, meant that assessments had to be based on oral or written (pen and paper) NP tests which have been shown to be associated with greater variability than computerised tests [53].

The control group was recruited opportunistically. The quality of RCI analyses is largely dependent upon how closely the control group matches the characteristics of the interest group. Barring randomisation, which would have been ideal yet unfeasible, we have undertaken careful matching to ensure that any significant differences in performance are not due to differences in age, education and oxygen saturation levels. It remains possible that there may be other unmeasured variables that may account for the effects such differences in levels of exercise between trekkers and control participants. Although none of NP tests were performed immediately after exercise or reaching designated ascent points so as to reduce possible proximal effects, differences between trekkers and controls were not directly controlled for. Although recruiting healthy participants was deemed the best technique to estimate practice effects, the small sample size of control group and potential differences in levels of exercise as result of the expedition may limit the accuracy of the calculation of practice effects.

Further work is required to elucidate the mechanisms of cognitive decline in conditions of acute hypoxia and to account for individual differences in cognitive performance and recovery rates.

### Authors’ translational perspective

The clinical neurocognitive effects of hypoxia manifest in diverse environments–from the agitated patient in the pre-hospital phase, to the deteriorating intensive care patient, to the elderly octogenarian developing a chest infection and concurrent confusion. By studying the neurocognitive effects of hypoxia on otherwise “healthy” individuals in a prospective manner, this study aimed to investigate this in a structured way. The results imply that, in some individuals at least, hypoxia results in a reduced ability to learn. This may link to disorientation in time and place that is commonly seen in hypoxic patients, particularly in critically ill patients. The notable predilection to loss of verbal ability may equate to inability to express oneself. Clinically it is often perceived that the elderly are more prone to confusion. In our study age was significantly associated with decline, and notably with delayed recovery (i.e., return back to the relative normoxia of Kathmandu). This suggests that cognitive recovery from hypoxia may persist longer in older people and this chimes with clinical experience of reduced cognitive resilience and delayed return to normal cognitive function in elderly patients exposed to hypoxia and other pathological stressors. Possibly of greatest importance is the marked heterogeneity of cognitive decline. Hypoxia did not have the same effect on everyone–some were more prone to neurocognitive decline than others. Further studies are needed to help identify biomarkers of predisposition and response for hypoxia-related cognitive impairment, particularly in older patients, in order to guide therapy and thereby minimise the magnitude and duration of impairment.

## Supporting information

S1 Fig Histogram of summary NP scores at EBC. (PNG)

S2 Fig Histogram of summary NP scores at Katmandu. (TIF)
