# Supplementary figures and images for: Caudwell Xtreme Everest: A prospective study of the effects of environmental hypoxia on cognitive functioning
Source: PLoS One. 2017 Mar 27;12(3):e0174277. doi: 10.1371/journal.pone.0174277 (PMC5367700; doi:10.1371/journal.pone.0174277)

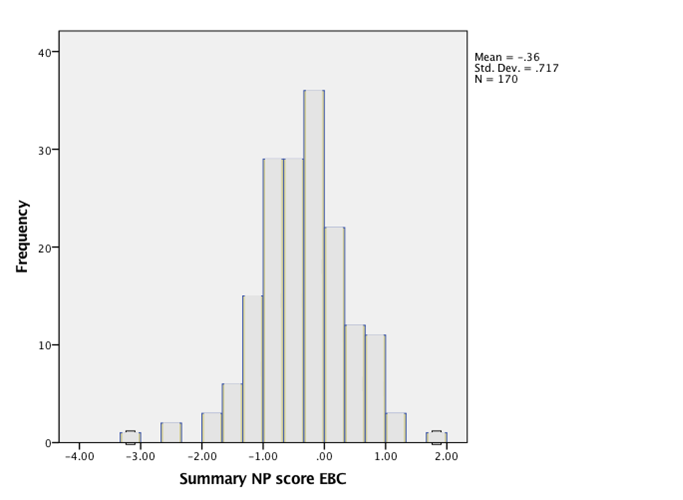

Supplement: S1 Fig — (PNG) [file pone.0174277.s001.png]

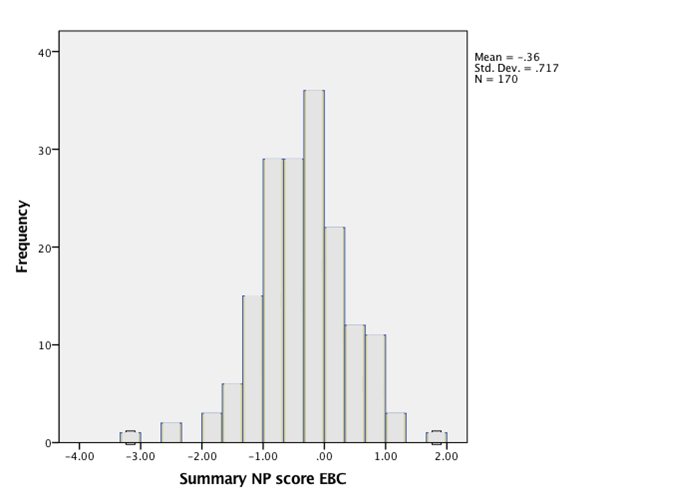

Supplement: S2 Fig — (TIF) [file pone.0174277.s002.tif]
